# Bicyclol Attenuates Acute Liver Injury by Activating Autophagy, Anti-Oxidative and Anti-Inflammatory Capabilities in Mice

**DOI:** 10.3389/fphar.2020.00463

**Published:** 2020-04-17

**Authors:** Tian-Ming Zhao, Ya Wang, You Deng, Xiao-Fei Fan, Xiao-Cang Cao, Li-Jun Hou, Li-Hong Mao, Lin Lin, Wei Zhao, Bang-Mao Wang, Kui Jiang, Jing-Wen Zhao, Chao Sun

**Affiliations:** ^1^ Department of Gastroenterology and Hepatology, Tianjin Medical University General Hospital, Tianjin, China; ^2^ Tianjin Institute of Digestive Disease, Tianjin Medical University General Hospital, Tianjin, China; ^3^ Department of Gastroenterology, Shanxi Academy of Medical Sciences Shanxi Bethune Hospital, Taiyuan, China; ^4^ Department of Gastroenterology, Tianjin Medical University General Hospital Airport Hospital, Tianjin, China

**Keywords:** bicyclol, autophagy, liver injury, inflammatory response, oxidative stress

## Abstract

Bicyclol, a novel synthetic antihepatitis drug, has been shown to protect against liver injury *via* various pharmacological activities. The purpose of the current study was to further investigate the protective effect of bicyclol against carbon tetrachloride (CCl_4_)-induced acute liver injury (ALI) and its underlying molecular mechanism, particularly autophagic machinery, anti-oxidative, and anti-inflammatory potentials. Our results found that treatment with bicyclol significantly reduced CCl_4_-induced hepatotoxicity by alleviating histopathological liver changes, decreasing the alanine transaminase levels, promoting autophagic flux, attenuating the expression of inflammatory cytokines, and modulating oxidative markers. Furthermore, bicyclol efficiently induced the conversion of LC3 and enhanced the liver expressions of ATG7 and Beclin-1. Meanwhile, bicyclol induced the activation of nuclear factor erythroid 2-related factor 2 (Nrf2) and p62. These protective effects may be mediated by activation of AMP-activated protein kinase and inhibition of mTOR or MAPK signaling pathways. Taken together, our study firstly suggests that bicyclol has protective potential against CCl_4_-induced hepatotoxicity, which might be closely associated with induction of autophagy, concomitant anti-oxidative stress, and anti-inflammatory response.

## Introduction

Acute liver injury (ALI) is associated with high mortality rates globally (Wu et al., 2010). The underlying molecular progress of ALI consists of a complicated interplay between oxidative stress, necrosis, apoptosis, and autophagy (Jaeschke et al., 2012; Dai et al., 2018). Carbon tetrachloride (CCl_4_)-induced ALI in rodent has been broadly used to investigate the therapeutic strategies due to its similarities with chemical liver damage in humans (Hamdy and El-Demerdash, 2012; Ma et al., 2014). Although it has been proved that the oxidative damage and inflammatory response play a critical role in this model (Tsai et al., 2017; Zhao et al., 2017), other molecular mechanisms are not fully understood.

Bicyclol, (4,4′-dimethoxy-5,6,5′,6′-bis [methylenedioxy]-2-hydroxymethyl-2′-methoxycarbonyl biphenyl), as an approved synthetic drug in China, has remarkably hepatoprotective effects and its core machinery may be relevant to scavenging free radicals and inhibiting oxidative stress with subsequent decrease in the toxicity of CCl_4_ intermediates to hepatocytes (Liu et al., 2005; Liu et al., 2017). More recently, it was reported that bicyclol has potent anti-proliferative activity and induces autophagy in human hepatoma cells via suppression of the PI3K/AKT and MEK/ERK pathways (Wang et al., 2016).

Autophagy is regarded as the main route for incorporation of cytoplasmic components into lysosomes. Autophagy comprises membrane biogenesis and formation of the autophagosome, which sequesters a region of cytosol and/or an entire organelle and subsequently fuses with the lysosome for degradation of its contents (Mizushima et al., 2008). Recently, modulation of the oxidative stress and autophagy crosstalk has been shown to be a new therapeutic target (Medvedev et al., 2017). Moreover, inflammation is often accompanied with formation of reactive oxygen species (ROS) and oxidative stress. Oxidative stress may accelerate the inflammation process by activating pro-inflammatory pathways, including mitogen-activated protein kinase (MAPK) and the well-known NOD-like receptor protein 3 (NLRP3) inflammasome pathways.

Given the above information, the purpose of this study is to investigate the protective effects of bicyclol against CCl_4_-induced ALI and decipher the molecular basis of this effect.

## Materials And Methods

### Chemicals and Reagents

CCl_4_ was purchased from Fuyu Chemical Industry Co., Ltd. (Tianjin, China), while 3-MA was purchased from Sigma-Aldrich (St. Louis, MO, USA). Bicyclol was given from the Beijing Union Pharmaceutical Company (Beijing, China) with purity over 99%.

### Animals

C57BL/6 mice (male, 6–8 weeks, 20–22 g) were purchased from National Institutes for Food and Drug Control (Beijing, China). Mice were housed in a room maintained at a temperature of 23 ± 2 °C and relative humidity of 50 ± 10% with a 12 h light-dark cycle. Mice were acclimatized for 1 week prior to experiment and had free access to food and water. All animal experiments were approved by the Institutional Animal Care and Use Committee at the Tianjin Medical University General Hospital.

The mice received an intraperitoneal (*i.p.*) injection of a mixture of CCl_4_ (50%) and oil (50%) at a dose of 2 ml/kg body weight. The control group was given an intraperitoneal injection of the same value of oil as the CCl_4_ group. In bicyclol treated group, mice were given bicyclol 200 mg/kg (suspended in 0.5% carboxymethyl cellulose) by gavage for three times in 1 day 1 h prior to CCl_4_ challenge, while other groups received an equal volume of vehicle. The dosage of bicyclol used was in agreement with previously published work (Dai et al., 2016). The mice were sacrificed at 24 and 48 h after the CCl_4_ injection. Thirty mice were randomly divided into five group as follows (n = 6 each group): (1) vehicle-treated normal control (control); (2) vehicle-treated CCl_4_ exposure at 24 h (CCl_4_ 24 h); (3) vehicle-treated CCl_4_ exposure at 48 h (CCl_4_ 48 h); (4) 200 mg/kg bicyclol-treated CCl_4_ exposure at 24 h (CCl_4_ + bicyclol 24 h); and (5) 200 mg/kg bicyclol-treated CCl_4_ exposure at 48 h (CCl_4_ + bicyclol 48 h).

### Cell Culture

Normal mouse hepatocytes AML 12 cell line was purchased from the Cell Bank of the Chinese Academy of Sciences (Shanghai, China) and cultured in DMEM/F12 medium with 10% fecal bovine serum in a 37°C with 5% CO_2_. Cells were additionally supplemented with 1% insulin-selenium-transferrin (Sigma-Aldrich, USA) and 40 ng/ml dexamethasone as recommended.

### Alanine Transaminase (ALT) Assay

The level of serum ALT was determined by using an Automated Chemical Analyzer (Hitachi 7080, Hitachi High-Technologies Corporation) with the standard diagnostic kits (Shanghai Kehua Bio-engineering Company, Shanghai, China).

### Hepatic Lipid Peroxidation Assay

Hepatic homogenates were analyzed for malondialdehyde (MDA) by measuring the level of thiobarbituric acid-reactive substances spectrophotometrically at 535 nm with 1,1,3,3-tetraethoxypropane (Sigma-Aldrich) as the standard.

### Histology and Immunohistochemistry

Liver tissue was collected 24 and 48 h after CCl_4_ treatment. A portion of liver tissue was fixed in 10% neutral buffered formalin for histology and immunohistochemistry, and the rest of the sample was used for real time-PCR and western blot analysis. Formalin-fixed, paraffin-embedded liver tissues were cut into 5 μm thickness sections and stained with hematoxylin and eosin (H&E). The Knodell score was used to grade the severity of the necroinflammatory process (Knodell et al., 1981).

### Transmission Electron Microscopy (TEM)

Liver tissues were fixed in 2.5% glutaraldehyde and 4% paraformaldehyde in 100 mM sodium phosphate (pH 7.2). Samples were washed with 100 mM Na cacodylate (pH 7.4), post-fixed in 2% osmium tetroxide and then washed again. The samples were dehydrated in a graded series of ethanol and propylene oxide and embedded in epoxy resin (TAAB 812 Resin; Marivac Industries, Montreal, QC, Canada). Ultrathin (60–70 nm) sections were counterstained with uranyl acetate and lead citrate and viewed using a Hitachi 7600 TEM (Hitachi High-Technologies America, Inc., Schaumburg, IL, USA) equipped with a Macrofire monochrome progressive scan CCD camera (Optronics, Inc., Muskogee, OK, USA) and AMTV image capture software (Advanced Microscopy Techniques, Corp., Danvers, MA, USA).

### Real-Time PCR Analysis

Total RNA was isolated from liver samples using Trizol reagent according to the manufacturer’s protocol. A total of 2.5 μg of RNA was reverse-transcribed into cDNA using SuperScript III First-Strand Synthesis System (Invitrogen, Carlsbad, CA, USA). Real-time PCR was performed using the DNA Engine with Chromo 4 Detector (MJ Research, Waltham, MA, USA). The following were added to a final reaction volume of 20 μl: 1x SuperMix (Platinum SYBR Green qPCR Kit; Invitrogen); cDNA (2 μl); and 0.5 μM of each primer. The amplification conditions were as follows: 50 °C (2 min); 95 °C (5 min); followed by 50 cycles of 95 °C (15 s) and 60 °C (30 s). The primers used are listed in **Table 1**.

**Table 1:** List of Primers for Real-time PCR.

Target	Gene ID	Primer	Sequence
P62	18412	FP	5′-GAGGCACCCCGAAACATGG-3′
		RP	5′-ACTTATAGCGAGTTCCCACCA-3′
Keap-1	50868	FP	5′-CAACTTCGCGGAGCAGATCG-3′
		RP	5′-AGCTGGCAGTGTGACAGGTT-3′
Nrf2	18024	FP	5′-CGAGATATACGCAGGAGAGGTAAGA-3′
		RP	5′-GCTCGACAATGTTCTCCAGCTT-3′
GSTA-1	14857	FP	5′-TGCCCAATCATTTCAGTCAG-3′
		RP	5′-CCAGAGCCATTCTCAACTA-3′
HO-1	15368	FP	5′-GAGCAGAACCAGCCTGAACTA-3′
		RP	5′-GGTACAAGGAAGCCATCACCA-3′
NQO-1	18104	FP	5′-TGGCCGAACACAAGAAGCTGGAA-3′
		RP	5′-CCCCGTGGACACCCTGAAGAGAGT-3′
NLRP3	216799	FP	5′-GTGGTGACCCTCTGTGAGGT-3′
		RP	5′-TCTTCCTGGAGCGCTTCTAA-3′
IL-1β	16176	FP	5′-GAAATGCCACCTTTTGACAGTG-3′
		RP	5′-TGGATGCTCTCATCAGGACAG-3′
IL-18	16173	FP	5′-GACTCTTGCGTCAACTTCAAGG-3′
		RP	5′-CAGGCTGTCTTTTGTCAACGA-3′
IL-6	16193	FP	5′-CCAGTTGCCTTCTTGGGACT-3′
		RP	5′-GGTCTGTTGGGAGTGGTATCC-3′
TNF-α	21926	FP	5′-CCACCACGCTCTTCTGTCTA-3′
		RP	5′-GGTTTGCTACGACGTGGGC-3′
GAPDH	14433	FP	5′-GGAGAAACCTGCCAAGTATG-3′
		RP	5′-TGGGAGTTGCTGTTGAAGTC-3′

FP, forward primer; RP, reverse primer.

### Western Blot Analysis

After the designated treatments were implemented, liver tissues or collected cells were lysed with RIPA buﬀer supplemented with protease inhibitors. The protein concentration was measured using the BCA protein assay kit. Total proteins (30 μg) were separated via 10% SDS-polyacrylamide gel electrophoresis (PAGE) and transferred to nitrocellulose (NC) membranes. The following primary antibodies were employed: primary rabbit antibodies against microtubule-associated protein 1 light chain 3 A/B (LC3 A/B) (1:1000, No. 12741), p62 (1:1000, No. 5114), Atg5 (1:1000, No. 12994), Atg7 (1:1000, No. 2631), Atg12 (1:1000, No. 4180), Beclin-1 (1:1000, No. 3738), nuclear factor erythroid 2-related factor 2 (Nrf2) (1:1000, No. 12721), mTOR (1:1000, No. 2983), phospho (p)-mTOR (1:1000, No. 5536), p38 (1:1000, No. 8690), p-p38 (1:1000, No. 4511), ERK (1:5000, No. 4696), p-ERK (1:2000, No. 4370), AMPK (1:1000, No. 5832), p-AMPK (1:1000, No. 2535), β-actin (1:1000, No. 4970), Lamin B (1:1000, No. 12255) (Cell Signaling Technology, Beverly, MA, USA), JNK (1:1000, ab208035), p-JNK (1:5000, ab76572), Kelch-Like ECH-Associated Protein 1 (Keap1) (1:1000, ab139729) (Abcam, Cambridge, MA, USA). Peroxidase-conjugated goat anti-rabbit or anti-mouse IgG (1:5000) (Zhongshan Golden Bridge Biotechnology, Beijing, China) were employed as the secondary antibodies. The specific protein bands were visualized using the enhanced western luminescent detection kit (Vigorous Biotechnology, Beijing, China). The results were quantified by densitometry using Image J software, and the densitometry results were normalized relative to the β-actin or Lamin B bands.

### Cytokine Measurement

Circulating cytokine profiles comprised mice from all experimental groups. For the cytokine assays, whole blood samples were collected into disposable vacuum blood collection tubes (BD, USA). After 0.5 h of standing in room temperature, and centrifuged at 2000 rpm/min for 10 min; serum was then obtained. The supernatant was pipetted in to EP tubes and stored at −80 °C until use. We quantitatively detected the expression level of four circulating cytokines, including interleukin (IL-1β), IL-6, IL-18 and tumor necrosis factor (TNF-α) using MILLIPLEX^®^ map Mouse High Sensitivity Cytokine Panels for 96-well assay (Millpore Corporation, Billerica MA, USA) on a Luminex platform. Only measurements with CV ≤20% were included in the analysis. All cytokine concentrations were analyzed in the same bead suspension to minimize interexperimental variability. For quality assurance, each sample was run twice, and the mean derivation was used as the index value.

### GFP-LC3 and RFP-GFP-LC3 Plasmid Transfection and Immunofluorescence

To observe autophagosome formation and autophagy flux, AML 12 cells were transiently transfected with the GFP-LC3 or GFP-RFP-LC3 expression plasmid (regularly kept in our lab) using Lipofectamine 3000, according to the manufacturer’s instructions.

### Statistical Analysis

All results are presented as means ± the standard deviation (SD). The overall significance of the data was examined by two-way analysis of variance. Differences between groups were considered statistically significant at p < 0.05 with the appropriate Bonferroni correction made for multiple comparisons.

## Results

### Bicyclol Treatment Mitigated CCl_4_-Induced ALI

**Figure 1:**
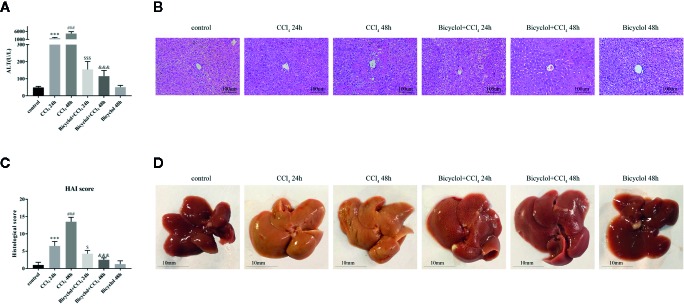
Effects of bicyclol on serum alanine transaminase (ALT) activity **(A)**, hematoxylin and eosin (H&E) staining **(B)**, histological score **(C)**, and macroscopic examination **(D)** at 24 and 48 h after CCl_4_ exposure. Mice were intraperitoneally injected a mixture of CCl_4_ (50%) and oil (50%) at a dose of 2 ml/kg body weight. Mice were given bicyclol 200 mg/kg by gavage for three times in 1 day 1 h prior to CCl_4_ challenge. Results are presented as mean ± SD for five mice per group. Significantly different (***p < 0.001, ^###^p < 0.001) from the control group. Significantly different (^$^p < 0.05, ^$$$^p < 0.001) from the CCl_4_ 24 h group. Significantly different (^&&&^p < 0.001) from the CCl_4_ 48 h group.

First, we assessed the time course of the hepatoprotective effect of bicyclol against CCl_4_-induced ALI. ALT levels in serum are acknowledged as key markers of hepatic damage, therefore the serum ALT of mice with ALI were detected. As shown in the **Figure 1A**, bicyclol treatment significantly reduced ALT levels in serum that was elevated by CCl_4_ compared to the control group.

Histological change of the livers of mice from CCl_4_ group indicated more apparent liver injury at 48 h, displayed as large proportion of extensive cellular necrosis with noticeably disturbed architecture and neutrophil infiltration (**Figure 1B**). Compared with the control group, the histological scores for CCl_4_ group at 24 and 48 h were increased to 6.5 ± 0.6 and 13.5 ± 0.6, respectively. Bicyclol treatment effectively mitigated the histological scores at 24 and 48 h to 4.3 ± 0.5 and 2.5 ± 0.3, respectively (**Figure 1C**). As presented in **Figure 1D**, these findings were also confirmed by macroscopic estimation.

### Bicyclol Treatment Enhanced Sustained Expression of Autophagy Protein in Mice With CCl_4_-Induced ALI

**Figure 2:**
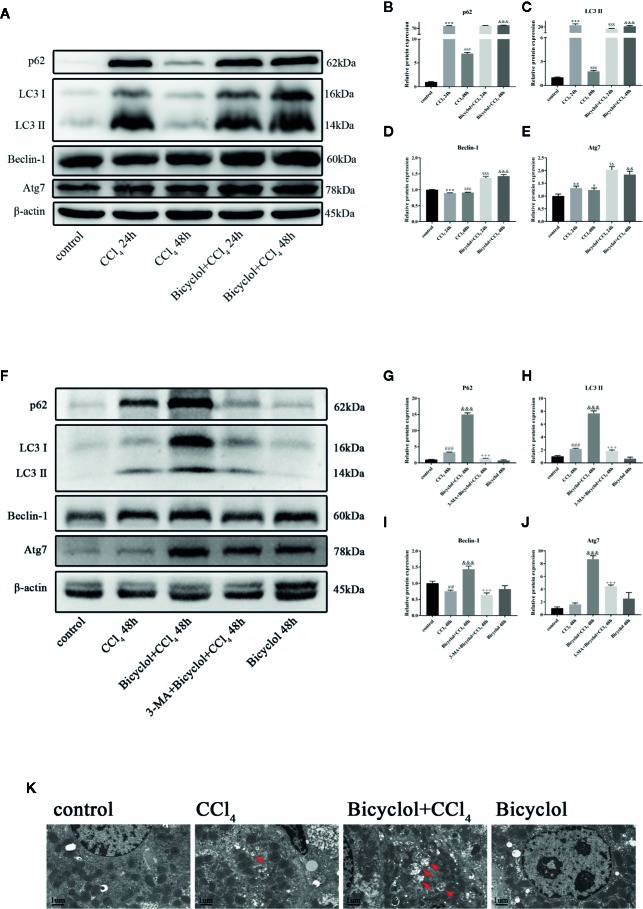
Effect of bicyclol and 3-MA on LC3-II, p62, ATG7, and Beclin-1 protein expressions **(A–J)** and transmission electron microscopy images **(K)** after CCl_4_ exposure. Mice were intraperitoneally injected a mixture of CCl_4_ (50%) and oil (50%) at a dose of 2 ml/kg body weight. Mice were given bicyclol 200 mg/kg by gavage for three times in 1 day 1 h prior to CCl_4_ challenge. Mice were pretreated with 3-MA before bicyclol. Results are presented as mean ± SD for five mice per group. Significantly different (**p < 0.01, ***p < 0.001, ^#^p < 0.05, ^###^p < 0.001) from the control group. Significantly different (^$$^p < 0.01, ^$$$^p < 0.001) from the CCl_4_ 24 h group. Significantly different (^&&^p < 0.01, ^&&&^p < 0.001) from the CCl_4_ 48 h group. Significantly different (^+++^p < 0.001) from the bicyclol + CCl_4_ 48 h group.

To evaluate autophagic machinery upon bicyclol treatment in CCl_4_-induced ALI, we examined changes of expression levels regarding LC3-II and p62 protein. The expression levels of LC3-II and p62 protein after 24 h of CCl_4_ challenged dramatically increased compared with that of the control group and returned to the control level after 48 h of CCl_4_ challenged (**Figure 2A**). However, treatment of bicyclol significantly increased the expression levels of LC3-II and p62 to 11.9-fold and 13.7-fold, respectively, compared with that of 48 h CCl_4_ challenged group (**Figures 2B, C**). Moreover, the expression levels of several pro-autophagy proteins, including Beclin-1 and Atg7, were significantly increased upon bicyclol treatment (**Figures 2D, E**). No significant changes were found with respect to protein expression of Atg5 and Atg12 (**Supplementary Figure**). In contrast, treatment with 3-MA (autophagy inhibitor) abrogated the elevated level of LC3-II, p62, Beclin-1, and ATG7 (**Figures 2F–J**). To confirm our western blot findings, we determined autophagic vacuoles, including autophagosomes and autolysosomes, by TEM (**Figure 2K**). The autophagic vacuoles manifested by double-membrane structures, encompassing undigested organelles and cytoplasm. The number of autophagic vacuole significantly increased in response to CCl_4_ challenge compared with the basal level in the control group, which was further augmented by bicyclol.

### Bicyclol Treatment Induced Autophagy in AML12 Cells

**Figure 3:**
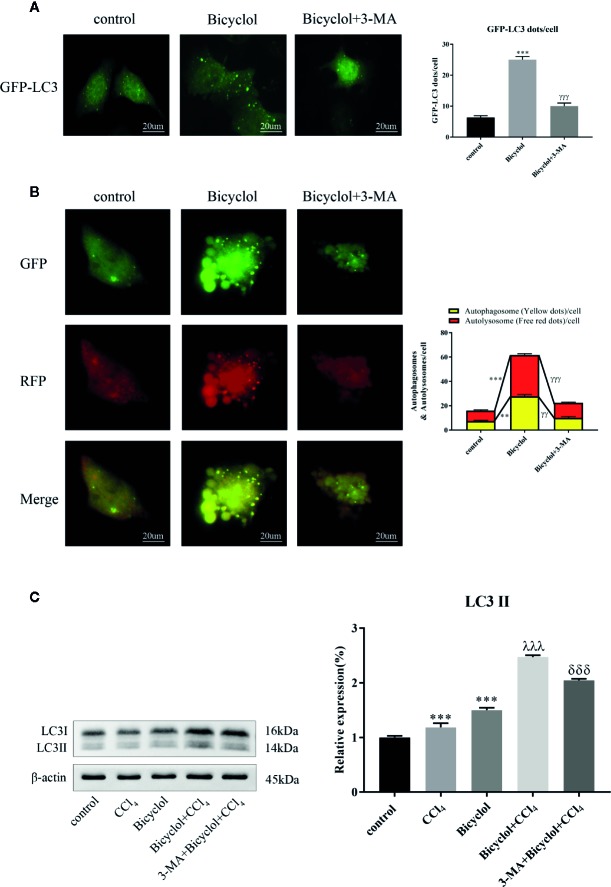
Effect of bicyclol and 3-MA on autophagic response in hepatocytes. AML12 normal mouse hepatocytes were pretreated with 3-MA (10 mmol/l) and then supplemented with bicyclol (500 μmol/l). **(A)** LC3 puncta formation was detected by transfecting cells with a GFP-LC3 plasmid, and bicyclol treatment significantly increased LC3 puncta formation. **(B)** A GFP-RFP dual-labeled LC3 plasmid was transfected to measure autophagic flux in cells. RFP- (red) and overlapped-positive areas (yellow) were significantly increased in bicyclol-treated AML12 cells compared with control. In the presence of 3-MA, the overlapped signals and red fluorescence in AML12 cells were both dramatically reduced. **(C)** LC3-II protein expression was detected by western blot analysis. 3-MA pretreatment decreased LC3-II accumulation, whereas bicyclol treatment significantly increased LC3-II levels in either the presence or the absence of CCl_4_ (2 μl/ml). Significantly different (**p < 0.01, ***p < 0.001) from the control group. Significantly different (^γγ^p < 0.01, ^γγγ^p < 0.001) from the bicyclol group. Significantly different (^λλλ^p < 0.001) from the CCl_4_ group. Significantly different (^δδδ^p < 0.001) from the bicyclol + CCl_4_ group.

Autophagy comprises early and late stages. The early stage is characterized by a formation of double-membrane bound vacuoles (autophagosomes), while there is a production of autolysosomes (via the fusion of autophagosomes with lysosomes) and lysosome-dependent degradation during the late stage. GFP-tagged LC3 plasmid transfection showed increased LC3 puncta formation after bicyclol treatment in AML12 cells (**Figure 3A**). To investigate autophagic flux, we transfected GFP-RFP dual-labeled LC3 into AML12 cells treated with bicyclol in the presence or absence of 3-MA to assess autophagosomes and autolysosomes. We found a clear increase in the overlapped signals (yellow) and RFP-LC3 (red) fluorescence in bicyclol-treated AML12 cells compared with controls, suggesting more autophagosomes and increased autolysosomes (**Figure 3B**). In the presence of 3-MA, the overlapped signals (yellow) and red fluorescence in AML12 cells were both dramatically reduced. As shown in **Figure 3C**, 3-MA pretreatment was expected to decrease LC3-II accumulation, whereas bicyclol treatment significantly increased LC3-II levels in either the presence or the absence of CCl_4_. Taken together, these results indicate that bicyclol can enhance autophagic response by promoting the formation and fusion of autophagic vesicles with lysosomes.

### Bicyclol Treatment Regulated the p62-Nrf2-Keap1 Pathway Followed by Increasing Downstream Antioxidant Enzymes

**Figure 4:**
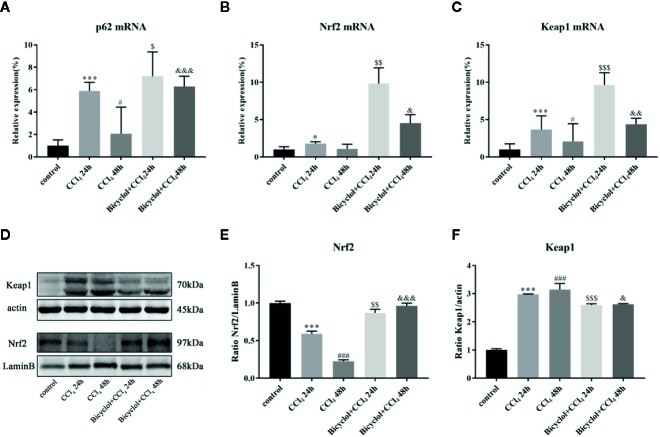
Effect of bicyclol on hepatic p62, Nrf2, and Keap1 mRNA expression **(A–C)** and Nrf2 and Keap1 protein expression in 24 and 48 h CCl_4_-triggered mice **(D–F)**. Mice were intraperitoneally injected a mixture of CCl_4_ (50%) and oil (50%) at a dose of 2 ml/kg body weight. Mice were given bicyclol 200 mg/kg by gavage for three times in 1 day 1 h prior to CCl_4_ challenge. Results are presented as mean ± SD for five mice per group. Significantly different (*p < 0.05, ***p < 0.001, ^#^p < 0.05, ^###^p < 0.001) from the control group. Significantly different (^$^p < 0.05, ^$$^p < 0.01, ^$$$^p < 0.001) from the CCl_4_ 24 h group. Significantly different (^&^p < 0.05, ^&&^p < 0.01, ^&&&^p < 0.001) from the CCl_4_ 48 h group.

Previous report has revealed that elevated p62 contributes to Nrf2 activation, which subsequently exhibits a strongly protective effect against hepatotoxicity accompanied with enhanced autophagy (Song et al., 2015). Having found transcriptional upregulation of p62 (both mRNA and protein levels) and enhancing autophagic process by bicyclol (**Figure 4A**), we then questioned if p62-Nrf2 interaction played any role in the current system. Our findings implicated that the bicyclol treatment efficiently increased mRNA level of Nrf2 and facilitated the nuclear transcription of Nrf2 (**Figures 4B-E**). It has been addressed that p62 can interact with Keap1, an adaptor of the Cul3-ubiquitin E3 ligase complex for degrading Nrf2, resulting in inhibition of Keap1-dependent Nrf2 degradation (Ichimura et al., 2013). Therefore we then asked if p62 accumulation led to Nrf2 activation by bicyclol via suppression of Keap1. As shown in **Figure 4F**, the expression level of Keap1 significantly increased 3.0-fold and 3.2-fold after 24 and 48 h of CCl_4_ exposure, respectively, from that of the control group. Moreover, treatment of bicyclol efficiently decreased the expression level of Keap1 to approximately 87.2% and 83.5% that of CCl_4_ exposure group after 24 and 48 h, respectively.

**Figure 5:**
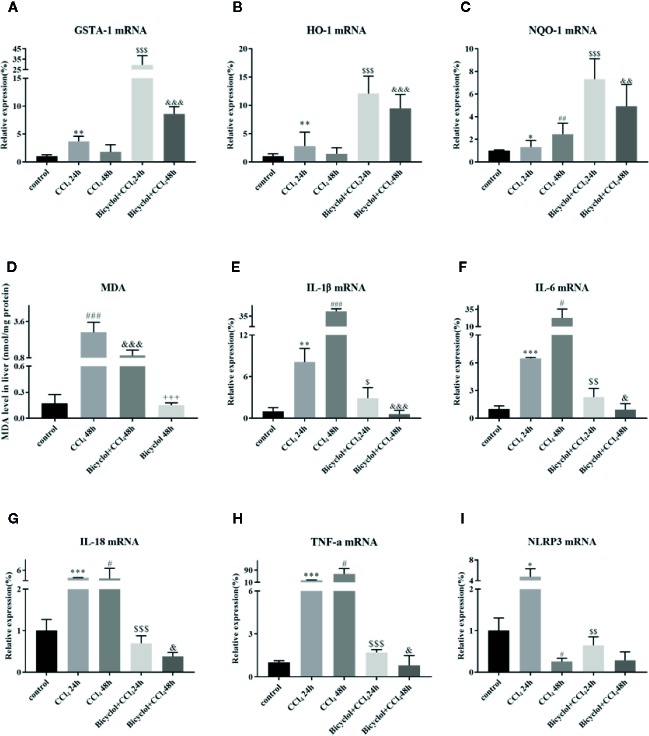
Effect of bicyclol on the hepatic antioxidant enzymes **(A–C)**, MDA **(D)**, and inflammatory cytokines/NLRP3 **(E–I)** expression levels after CCl_4_ exposure. Mice were intraperitoneally injected a mixture of CCl_4_ (50%) and oil (50%) at a dose of 2 ml/kg body weight. Mice were given bicyclol 200 mg/kg by gavage for three times in 1 day 1 h prior to CCl_4_ challenge. Results are presented as mean ± SD for five mice per group. Significantly different (*p < 0.05, **p < 0.01, ***p < 0.001, ^#^p < 0.05, ^##^p < 0.01, ^###^p < 0.001) from the control group. Significantly different (^$^p < 0.05, ^$$^p < 0.01, ^$$$^p < 0.001) from the CCl_4_ 24 h group. Significantly different (^&^p < 0.05, ^&&^p < 0.01, ^&&&^p < 0.001) from the CCl_4_ 48 h group. Significantly different (^+++^p < 0.001) from the bicyclol + CCl_4_ 48 h group.

Growing evidence implies that activation of Nrf2 signaling and the upregulation of downstream antioxidant enzymes are critical to suppress oxidative stress and maintain the cellular homeostasis (De Vries et al., 2008). In light of this, we investigated whether bicyclol actives GSTA-1, HO-1, and NQO-1 expression through Nrf2 pathway. HO-1 is a cytoprotective endogenous enzyme, which encompasses both anti-inflammatory and anti-oxidative effects by catalyzing the first and rate limiting step in the catabolism of the prooxidant heme to carbon monoxide, biliverdin, and free iron (Klaassen and Reisman, 2010). NQO-1, a cytosolic flavoprotein, catalyzes two-electron reduction and detoxification of quinones and other redox cycling exogenous and endogenous chemicals (Vasiliou et al., 2006). In the current study, the mRNA expression levels of GSTA-1, HO-1, and NQO-1 exhibited significant increases in bicyclol group (**Figures 5A–C**). Intriguingly, CCl_4_ group also showed the upregulation of these three genes expressions, which is consistent with previous study (Choi et al., 2011; Su et al., 2015). Moreover, the hepatic MDA level in liver tissue was significantly enhanced compared with that of the control group, but bicyclol treatment dramatically recovered this disordered change in the liver due to its antioxidant activity (**Figure 5D**).

### Bicyclol Treatment Inhibited CCl_4_-Induced Cytokines Secretion and NLRP3 Inflammasome Expression in Mice

Given CCl_4_ could result in liver injury through extensive inflammatory response (Zhao et al., 2017), several inflammatory cytokines were determined in livers of mice that were CCl_4_-challenged using real-time PCR and Milliplex. As shown in **Figure 5** and **Table 2**, CCl_4_ noticeably stimulated the mRNA expression and serum levels of IL-1β, IL-6, IL-18, and TNF-α compared to the control group, whereas bicyclol treatment decreased these inflammatory cytokine production, suggesting that bicyclol retained effective anti-inflammatory activity. Furthermore, previous report implicated that CCl_4_-induced ALI can be alleviated by inhibiting NLRP3 inflammasome activation through modulating Nrf2 anti-oxidant signaling pathway (Shi et al., 2018). Interestingly, bicyclol treatment also dramatically suppressed the expression of NLRP3 at 24 h (**Figure 5I**).

**Table 2:** Effect of bicyclol on serum IL-1β, IL-6, IL-18, and TNF-α levels in CCl_4_-treated mice.

Group	IL-1β (pg/ml)	IL-6 (pg/ml)	IL-18 (pg/ml)	TNF-α (pg/ml)
Control	12.8 ± 3.5	26.8 ± 3.8	20.6 ± 2.1	6.97 ± 1.44
CCl_4_ 24 h	27.0 ± 3.4^a^	47.9 ± 2.2^a^	41.9 ± 3.2^a^	75.7 ± 9.6^b^
CCl_4_ 48 h	41.9 ± 5.0^b^	96.1 ± 10.0^b^	66.4 ± 6.3^b^	94.2 ± 6.5^b^
CCl_4_ + bicyclol 24 h	20.4 ± 1.5	28.6 ± 3.2^c^	31.9 ± 1.6	26.5 ± 3.9^d^
CCl_4_ + bicyclol 48 h	12.1 ± 2.5^d^	24.5 ± 2.8^d^	22.2 ± 7.6^d^	8.53 ± 1.60^d^

Results are presented as mean ± SD of six mice per group. In bicyclol treated group, mice were given bicyclol 200 mg/kg by gavage for three times in 1 day 1 h prior to CCl_4_ challenge.

a p < 0.01 significantly different from the control group.

b p < 0.001 significantly different from the control group.

c p < 0.01 significantly different from the CCl_4_-treated group at the same time-point.

d p < 0.001 significantly different from the CCl_4_-treated group at the same time-point.

### Bicyclol Treatment Regulated CCl_4_-Induced AMPK, -mTOR, and -MAPK Signaling Pathway in Mice With ALI

**Figure 6:**
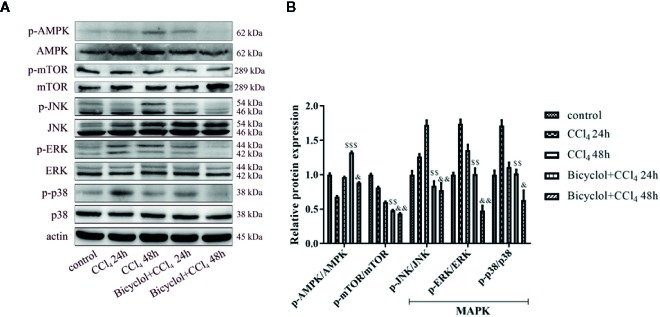
Effect of bicyclol on hepatic AMPK, mTOR, and MAPK protein expressions at 24 and 48 h after CCl_4_ exposure. Mice were intraperitoneally injected a mixture of CCl_4_ (50%) and oil (50%) at a dose of 2 ml/kg body weight. Mice were given bicyclol 200 mg/kg by gavage for three times in 1 day 1 h prior to CCl_4_ challenge. Results are presented as mean ± SD for five mice per group. Significantly different (^$$^p < 0.01, ^$$$^p < 0.001) from the CCl_4_ 24 h group. Significantly different (^&^p < 0.05, ^&&^p < 0.01) from the CCl_4_ 48 h group.

Since the AMP-activated protein kinase (AMPK) and mTOR play an essential role in the transcriptional regulation of autophagy (Mihaylova and Shaw, 2011; Sui et al., 2014), we investigated the phosphorylation status of both proteins in CCl_4_-triggered ALI. As shown in **Figure 6**, the level of p-AMPK/AMPK protein expression decreased to 68.9% of the control at 24 h CCl_4_ challenged, whereas bicyclol reversed this effect by increasing 1.9-fold over the CCl_4_ exposure group. Moreover, in the CCl_4_ + bicyclol 24 h group, the expression level of p-mTOR/mTOR decreased to approximately 48.3% and 59.3% that of the control and CCl_4_ exposure group, respectively. In the CCl_4_ + bicyclol 48 h group, the expression level of p-mTOR/mTOR decreased to approximately 43.6% and 72.3% that of the control and CCl_4_ exposure group, respectively. The MAPK, including the c-Jun NH2-terminal kinase (JNK), ERK, and p38, has been considered the main signaling pathway relevant to acute inflammation, which is also involved in the process of autophagy and anti-oxidant activities (Song et al., 2015; Lv et al., 2018; Lv et al., 2019). Our results showed that respective level of p-JNK/JNK protein expression increased 1.7-fold at 48 h, p-ERK/ERK and p-p38/p38 increased 1.7-fold and 1.7-fold at 24 h CCl_4_ exposure, but bicyclol treatment effectively inhibited MAPK phosphorylation, indicating multiple responses and pathways may be responsible for bicyclol-mediated hepatoprotective effect.

## Discussion

**Figure 7:**
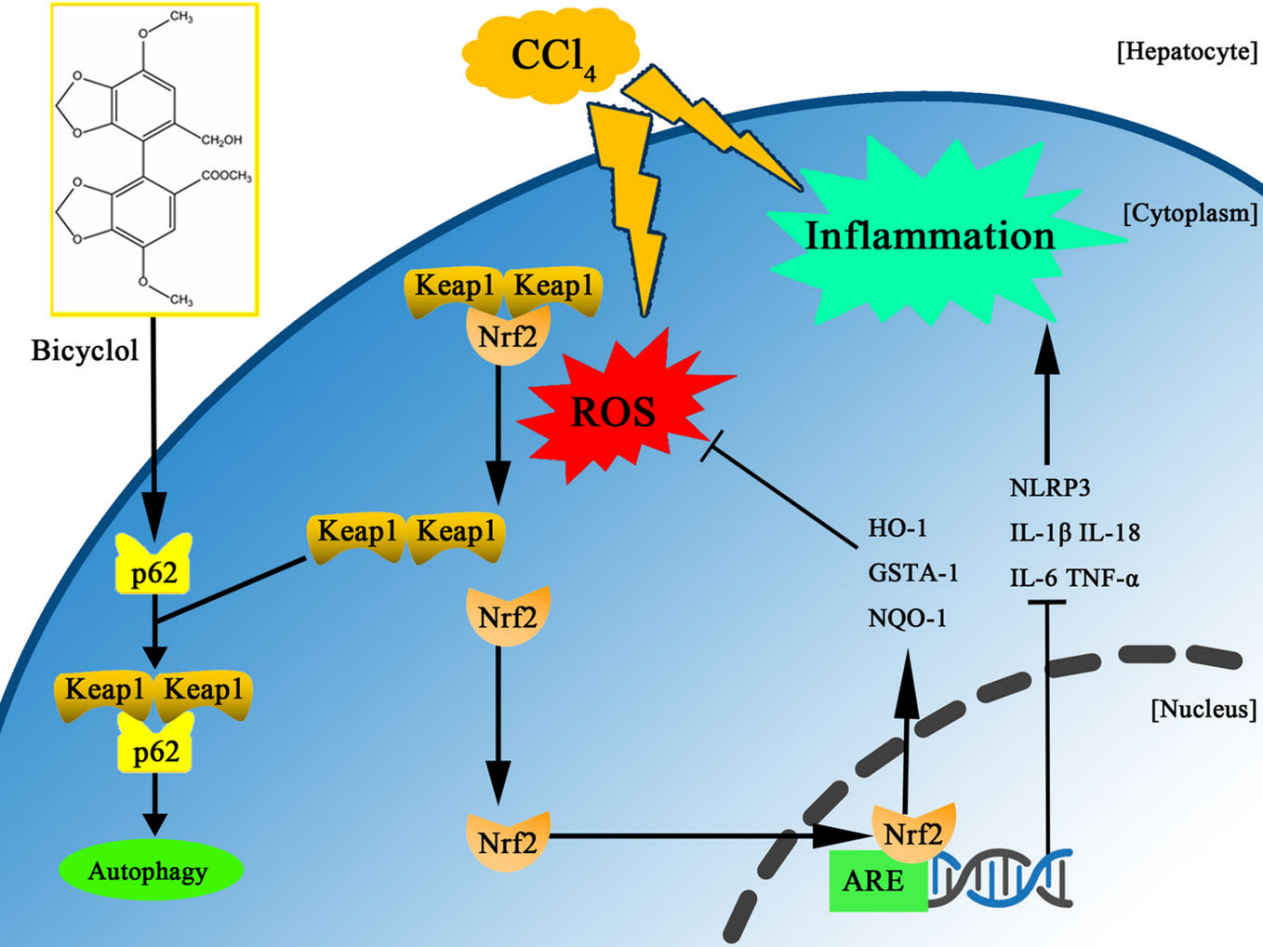
Scheme of the protective effects of bicyclol on CCl_4_-induced acute liver injury. Bicyclol possesses its impact *via* autophagy induction, inhibition of oxidative stress, and NLRP3 inflammasome inactivation, mainly relying on p62-Nrf2-Keap1 pathway.

Bicyclol has been widely used in the clinic to treat patients with chronic HBV infection via interruption of virus replication and improvement of liver function (Zhao and Liu, 2001; Wang and Li, 2006; Liu, 2009). More recently, it was reported that bicyclol effectively suppressed inflammatory response by reversing HCV-disturbed mitochondrial transmembrane potential (Li et al., 2018). Intriguingly, Wang et al. also demonstrated that bicyclol exhibited potent anti-proliferative activity in hepatoma cells (Wang et al., 2016). However, it remains to be clarified whether bicyclol has a potential against CCl_4_-triggered ALI by interfering with autophagic machinery, inflammatory response, and oxidative stress. In this study, we found for the first time that bicyclol could create a protective effect by induction of autophagy, inhibition of oxidative stress, and NLRP3 inflammasome, mainly relying on p62-Nrf2-Keap1 signaling pathway (**Figure 7**).

A growing body of literature indicates that regulation of autophagy may affect the progression of liver damage. Autophagy plays a pivotal role in cell survival as well as the modification of cell death, which is essential for maintenance of liver function (Ueno and Komatsu, 2017). Deficiency in autophagy promotes inflammatory response and oxidative stress, ultimately leading to a variety of diseases (Swanson and Molofsky, 2005; Scherz-Shouval et al., 2007). Previous studies have reported that autophagic flux is impaired in response to CCl_4_ challenge (Wang, 2015; Dai et al., 2018). Accordingly, our results showed that LC3-II protein expression remarkably increased 24 h after CCl_4_ challenged and declined by 48 h, suggesting autophagy induced by CCl_4_ acted as a cellular adaption mechanism and was activated in a transient manner. Furthermore, bicyclol augmented this effect at 48 h, which is less pronounced at 24 h after CCl_4_ exposure. This pattern was similar to the results of serum ALT activity and histological score, suggesting that bicyclol therapy enhanced adaptive autophagy in CCl_4_-induced ALI, converting it from a transient response to a persistent activation (Yan et al., 2018). Importantly, in the presence of 3-MA (an autophagy inhibitor blocks autophagosome formation by interfering with the activity of VPS34), the increase of LC3-II and p62 induced by bicyclol was substantially abrogated and the hepatic protection conferred by bicyclol was abolished.

In this study, bicyclol treatment also augmented the expression level of other autophagy-related proteins including ATG7 and Beclin-1. Specially, ATG7 is a key factor in the ubiquitin-like pathway of LC3 lipidation, while Beclin-1 interacts with VPS34, HMGB1 and Rubicon for modulating the autophagy process (Itakura and Mizushima, 2010; Shi et al., 2017). Furthermore, LC3-II and Beclin-1 are markers of autophagic flux since they involve in the initiation and closure of the autophagic vesicle, respectively (Itakura and Mizushima, 2010). Additionally, TEM images represented that bicyclol increased the number of autophagic vacuoles, and autophagic flux was promoted by bicyclol as indicated by the increase in autophagosomes and autolysosomes in AML12 cells. Collectively, we believed that bicyclol contributes to autophagy *in vivo* and *in vitro*.

Another novel pharmacological activity of bicyclol is its potential against CCl_4_-triggered hepatotoxicity, which is mediated by p62-Nrf2-Keap1 axis. P62 is a substrate of autophagy, whose biological function remains controversial. Under normal status, p62 recognizes cellular waste, including invading pathogens, damaged organelles, and aggregate-prone proteins, which is then cleared by autophagy (Klionsky et al., 2008). However, p62 accumulation with aggregates of ubiquitylated proteins, under the condition of disrupted autophagy, can result in various pathological consequences including liver diseases (Rusten and Stenmark, 2010; Takamura et al., 2011). It has been reported that p62 induction is beneficial in protecting and preventing against alcohol-triggered liver injury under conditions of autophagy sufficiency, which is probably related to Keap1 degradation-dependent activation of Nrf2 (Song et al., 2015). Intriguingly, our results also demonstrate that p62 serves as a positive regulator of Nrf2-Keap1 pathway, which is upregulated via a transcriptional mechanism (both in mRNA and protein level) rather than as the result of impaired autophagic machinery. One plausible interpretation of this p62 dependency in the bicyclol-induced autophagic Keap1 degradation is that p62 acts with LC3 through its LC3-interacting region and gives rise to the formation of a tertiary complex, p62-Keap1-LC3, which engages in the autophagy process. As a result, p62 elicits the transcriptional expression of Nrf2 target genes, including GSTA-1, HO-1, and NQO-1, and prevents CCl_4_-exposure mediated liver damage.

It is known that the hepatic metabolism of CCl_4_ releases extensive ROS, which in turn leads to autophagy, inflammation, and tissue necrosis (Xie et al., 2015; Shi et al., 2017; Wang et al., 2018). In addition, oxidative stress also contributes to inflammation by activating NLRP3 inflammasome. In this regard, Nrf2 is a key transcription factor that is essential for attenuating inflammation- and oxidative stress-associated diseases. Previous report has shown that the activation of Nrf2 not only rescues the tissues from oxidative damage but also exhibits a protective potential against inflammation in the pathogenesis of liver damage both *in vivo* and *in vitro* (Jia et al., 2018). Our results uncovered that bicyclol treatment dramatically inhibited IL-1β, IL-6, IL-18, and TNF-α generation and alleviated NLRP3 and MDA production.

The modulation of autophagy by bicyclol in liver damage is a novel finding, yet the need to identify the signaling pathway through which bicyclol triggers autophagy remains. Accumulating evidence implies that autophagy can be regulated by mTOR and MAPK (Chung et al., 2017; Zhang et al., 2017). The MAPK, including JNK, ERK, and p38, results in the transcription of genes contributing to cellular response to a plethora of stimuli such as proinflammatory mediators (Marino et al., 2014; Dai et al., 2018). It has also been known that activation of AMPK inhibits mTOR signaling pathway (Inoki et al., 2003). In the current study, the expression of p-JNK, p-ERK, and p-p38 exhibited dynamic changes during 48 h after CCl_4_ exposure. In this regard, we observed a dramatic increase in the expression of p-AMPK in the early phase of CCl_4_-induced ALI (i.e., at 24 h) upon bicyclol treatment, which was accompanied with a significant decrease in the expression of p-mTOR, p-JNK, p-ERK, as well as p-p38. Taken together, these data suggest that modulation of AMPK-mTOR and MAPK activities are involved in the hepatoprotection of bicyclol.

## Conclusion

In conclusion, this study demonstrated bicyclol has protective potential against CCl_4_-induced hepatotoxicity, which might be closely associated with induction of autophagy, concomitant anti-oxidative stress and anti-inflammatory response.

## Data Availability Statement

The datasets generated for this study are available on request to the corresponding authors.

## Ethics Statement

The animal study was reviewed and approved by Institutional Animal Care and Use Committee at the Tianjin Medical University General Hospital.

## Author Contributions

T-MZ, YW, and Y-D designed and performed the experiments, analyzed the data and wrote the manuscript. X-FF, X-CC, L-JH, L-HM, and LL performed the experiments and analyzed the data. WZ, B-MW, and KJ performed the experiments. J-WZ and CS oversaw the project, designed the experiments, analyzed the data, and wrote the manuscript.

## Funding

This work is partly supported by Tianjin Research Innovation Project for Postgraduate Students (2019YJSS186) to T-MZ.

## Conflict of Interest

The authors declare that the research was conducted in the absence of any commercial or financial relationships that could be construed as a potential conflict of interest.

## Abbreviations

CCl_4_, carbon tetrachloride; ALI, acute liver injury; 3-MA, 3-methyladenine; ALT, alanine transaminase; H&E, hematoxylin and eosin; TEM, transmission electron microscopy; SD, standard deviation.
