# Supplementary figures and images for: Bicyclol Attenuates Acute Liver Injury by Activating Autophagy, Anti-Oxidative and Anti-Inflammatory Capabilities in Mice
Source: Front Pharmacol. 2020 Apr 17;11:463. doi: 10.3389/fphar.2020.00463 (PMC7181473; doi:10.3389/fphar.2020.00463)

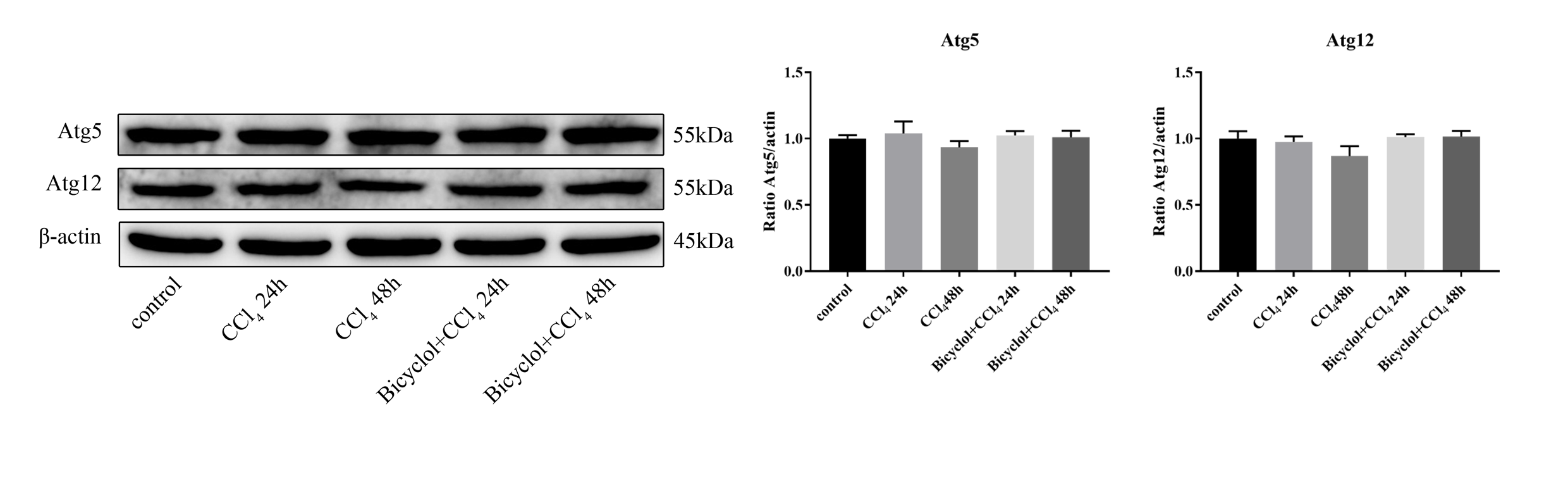

Supplement: Supplementary file 1 [file Image_1.tif]
